# Population Substructures of *Castanopsis tribuloides* in Northern Thailand Revealed Using Autosomal STR Variations

**DOI:** 10.3390/plants14152306

**Published:** 2025-07-26

**Authors:** Patcharawadee Thongkumkoon, Jatupol Kampuansai, Maneesawan Dansawan, Pimonrat Tiansawat, Nuttapol Noirungsee, Kittiyut Punchay, Nuttaluck Khamyong, Prasit Wangpakapattanawong

**Affiliations:** 1 Center of Multidisciplinary Technology for Advanced Medicine (CMUTEAM), Faculty of Medicine, Chiang Mai University, Chiang Mai 50200, Thailand; patcharawadee.t@cmu.ac.th; 2 Department of Biology, Faculty of Science, Chiang Mai University, Chiang Mai 50200, Thailand; jatupol.k@cmu.ac.th (J.K.); queendansawan@gmail.com (M.D.); pimonrat.t@cmu.ac.th (P.T.); nuttapol.n@cmu.ac.th (N.N.); lord_tack@hotmail.com (N.K.); 3 Forest Restoration Research Unit, Department of Biology, Faculty of Science, Chiang Mai University, Chiang Mai 50200, Thailand; 4 Queen Sirikit Botanic Garden, The Botanical Garden Organization, Chiang Mai 50180, Thailand; punchay.botany@outlook.com

**Keywords:** genetic diversity, population structure, STR markers, *Castanopsis tribuloides*, forest conservation, Thailand

## Abstract

This study investigates the genetic diversity and population structure of *Castanopsis tribuloides*, a vital tree species in Asian forest ecosystems. Understanding the genetic patterns of keystone forest species provides critical insights into forest resilience and ecosystem function and informs conservation strategies. We analyzed population samples collected from three distinct locations within Doi Suthep Mountain in northern Thailand using Short Tandem Repeat (STR) markers to assess both intra- and inter-population genetic relationships. DNA was extracted from leaf samples and analyzed using a panel of polymorphic microsatellite loci specifically optimized for *Castanopsis* species. Statistical analyses included the assessment of forensic parameters (number of alleles, observed and expected heterozygosity, gene diversity, polymorphic information content), population differentiation metrics (G_ST_), inbreeding coefficients (F_IS_), and gene flow estimates (N_m_). We further examined population history through bottleneck analysis using three models (IAM, SMM, and TPM) and visualized genetic relationships through principal coordinate analysis and cluster analysis. Our results revealed significant patterns of genetic structuring across the sampled populations, with genetic distance metrics showing statistically significant differentiation between certain population pairs. The PCA and cluster analyses confirmed distinct population groupings that correspond to geographic distribution patterns. These findings provide the first comprehensive assessment of *C. tribuloides* population genetics in this region, establishing baseline data for monitoring genetic diversity and informing conservation strategies. This research contributes to our understanding of how landscape features and ecological factors shape genetic diversity patterns in essential forest tree species, with implications for managing forest genetic resources in the face of environmental change.

## 1. Introduction

*Castanopsis tribuloides* (Sm.) A.DC. is a native tree species found in northern Thailand, including Doi Suthep Mountain in Chiang Mai, and is commonly planted in restoration areas such as Ban Mae Sa Mai in the Mae Rim district [1]. This species belongs to the Fagaceae family, a large group of evergreen tropical trees that thrive in mixed evergreen/deciduous forests, pine forests, and other wet, tropical hill forests across regions like India, Thailand, Myanmar, and Indochina. In Thailand, Fagaceae includes genera such as *Castanopsis*, *Lithocarpus*, *Quercus*, and *Trigonobalanus* [2,3]. Among these, *Trigonobalanus doichangensis* is a rare and endangered species [4]. *Castanopsis* species exhibit a predominantly insect-mediated pollination system [5]. Their trees bear unisexual flowers arranged on spikes or catkins, with male and female flowers often appearing on the same individual (monoecious) or rarely on separate individuals. In *Castanopsis sieboldii*, for example, effective pollen dispersal is primarily facilitated by insects, particularly bees, with pollen flow patterns strongly influenced by the spatial arrangement and density of adult trees. Although *Castanopsis* has the potential for long-distance pollen dispersal, most pollination events occur over short distances, leading to spatially limited gene flow in dense populations. This insect-pollinated system helps maintain genetic diversity within populations, as pollen from neighboring trees is the main contributor to the genetic makeup of offspring, though occasional long-distance pollen flow can introduce additional diversity [6]. Fagaceae species are a key selection for restoration efforts by the Forest Restoration Research Unit of Chiang Mai University (FORRU-CMU), which was established in 1994. These species are chosen for their fast growth, ability to suppress weeds, resilience to fire, and role in attracting seed-dispersing wildlife [1]. Commonly used for restoration in degraded areas of northern Thailand, other native species planted include those from the Fabaceae, Fagaceae, and Moraceae families, with *Ficus* species being particularly prominent [1]. *C. tribuloides* is a key framework tree species identified for planting in these degraded areas, with seeds typically collected from nearby natural forests. On Doi Suthep Mountain, *C. tribuloides* is found at elevations above 1000 m above sea level.

Genetic diversity and population structure of plant species represent an essential indicator of forest functions, resilient ecosystems, successional forest restoration, and the evolution of plant species. Moreover, molecular population genetics has been studied for levels of genetic variation for an understanding of genome-wide patterns and genomic resources to be combined with phenotypic variations for genetic conservation [7]. Genetic information can be explored using molecular markers, including Random Amplified Polymorphic DNA (RAPD), Amplified Fragment Length Polymorphism (AFLP), and Short Tandem Repeats (STRs) or microsatellite markers [8]. STR markers are used to study genetic diversity and population structure in plant, animal, and human populations. Moreover, STR primers are developed and can be applied to be used with the nearest species [9].

The genetic diversity of *C. sieboldii* and *C. cuspidata* in a natural forest in Japan reveals high genetic variation and low genetic differentiation among populations, with a notable inbreeding coefficient (F_IS_) that could lead to population substructure in the area [10]. A study on the population structure and genetic traits of *C. fargesii* in a natural forest in China showed high genetic diversity, moderate genetic differentiation among populations, extensive gene flow between groups, and low levels of inbreeding [9]. Additionally, genetic analysis of *C. eyrei* populations in China indicated low genetic differentiation, with higher genetic diversity observed in medium-altitude populations compared to those at lower or higher altitudes [11].

Zeng et al. [12] investigated whether *Castanopsis × kuchugouzhui* is a natural hybrid between *C. sclerophylla* and *C. tibetana* using chloroplast DNA sequences and 29 nuclear STR markers. Chloroplast haplotype analysis revealed that *C. × kuchugouzhui* shared haplotype H2 with *C. sclerophylla*, while STRUCTURE (Version 2.3.4) analysis identified two distinct genetic pools corresponding to the parent species and showed genetic admixture in *C. × kuchugouzhui*. The NewHybrids analysis further confirmed that *C. × kuchugouzhui* is an F2 hybrid between *C. sclerophylla* and *C. tibetana*, validating its hybrid status. Wu et al. [13] investigated genetic differentiation among three *Castanopsis* species using 15 nuclear STR markers across 308 individuals from 17 populations. The research revealed moderate but significant genetic differentiation among mainland *C. chinensis* and the Hainan Island species *C. qiongbeiensis* and *C. glabrifolia*, with greater differentiation between *C. chinensis* and *C. glabrifolia*. Demographic simulations showed unidirectional gene flow from mainland to island, reflecting historical land bridges between Hainan and mainland China.

A study of *C. tribuloides* annually planted in Ban Mae Sa Mai, Chiang Mai, Thailand, by FORRU-CMU revealed high genetic diversity and low inbreeding. This outcome demonstrates the effectiveness of their seed collection method, which emphasizes gathering a broad range of seeds [14]. Traditionally, seedlings for restoration are sourced from seeds collected from mature trees in natural forests, grown in nurseries, and then planted in restoration sites. Maintaining genetic diversity and understanding population structure within these natural forests is crucial, as they serve as the primary genetic resource for restoration efforts. To ensure the long-term health and resilience of plant populations, forest management and conservation strategies should prioritize the preservation of genetic diversity. This includes optimizing restoration programs, establishing seed banks, designating specific protected areas, and focusing on the conservation of endangered species. To optimize seed collection for future forest restoration initiatives, this study examined the genetic information and population structure of *C. tribuloides* within Doi Suthep Mountain, Chiang Mai, Thailand. We sought to delineate subpopulations based on altitude and genetic diversity, thereby improving our understanding of the species’ genetic resources.

## 2. Materials and Methods

### 2.1. Sample Collection

*C. tribuloides*, a native tree species, is found on Doi Suthep Mountain, Chiang Mai, Thailand. For this study, samples were collected from three plots within Doi Suthep Mountain. Plot I (low elevation) was located at an elevation from 1074 to 1149 m above sea level, Plot II (mid elevation) from 1386 to 1469 m, and Plot III (high elevation) from 1572 to 1668 m. We collected young leaf samples and GPS coordinates from a total of 84 adult trees. The leaf samples were washed, dried, placed in labeled zip-lock bags, and stored at −20 °C for subsequent DNA extraction.

### 2.2. DNA Extraction

Leaf samples were cut into small pieces and then ground using liquid nitrogen. Genomic DNA was extracted with the PureDireX Plant Genomic DNA Isolation Kit (Bio-Helix, Keelung City, Taiwan). DNA quantification and quality assessment were performed using a NanoDrop^®^ spectrophotometer and 1.5% agarose gel electrophoresis. The extracted genomic DNA was diluted in dH_2_O to a final concentration of 50 ng/µL and stored at 4 °C.

### 2.3. Molecular Marker for Studying Genetic Information

Eleven anonymous STR primers, originally developed from the whole-genome sequence data of *C. tribuloides* [9] (Table 1), were used to analyze the genetic information of Fagaceae in this study. We initially screened these primers using four samples from each plot, selecting those exhibiting high polymorphism. Subsequently, the forward primers of these polymorphic loci were fluorescently labeled with 6FAM, PET, HEX, and TAMRA [15]. PCR amplification was performed under the following conditions: initial denaturation at 94 °C for 5 min, followed by 35 cycles of 94 °C for 20 s, 55 °C for 1 min, and 60 °C for 1 min, with a final extension at 60 °C for 5 min. The annealing temperature ranged from 55 °C to 62 °C. PCR product sizes were determined through fragment analysis using Applied Biosystems 3730 × l DNA analyzer (96-capillary) at the Germplasm Bank of Wild Species, Kunming Institute of Botany, Chinese Academy of Sciences, China. Allele sizes for each primer were then estimated using GeneMarker^®^, a genotype software version 2.6.4 (Soft Genetics LLC, State College, PA, USA). STR allele sizes were assigned by comparing peak positions in each sample to these reference allele sizes, and the resulting data were recorded in a spreadsheet [16]. Reference allele sizes were derived from the same individual samples for each primer.

### 2.4. Statistical Analysis

Forensic parameters of each STR locus were analyzed using various computational tools to assess allele frequencies and genetic diversity. Arlequin v.3.5.2.2 [17] was employed to compute allele frequencies, Hardy–Weinberg equilibrium (HWE) *p*-values, observed heterozygosity (H_O_), expected heterozygosity (H_E_), the total number of alleles, and gene diversity (GD). To ensure statistical robustness, significance levels for HWE were adjusted using the sequential Bonferroni correction (α = 0.05/11). Additionally, forensic parameters were determined using the Excel PowerStats spreadsheet [18], which included power of discrimination (PD), matching probability (MP), polymorphic information content (PIC), power of exclusion (PE), and typical paternity index (TPI). Furthermore, combined forensic metrics such as combined power of discrimination (CPD), combined matching probability (CMP), and combined power of exclusion (CPE) were also calculated.

The intra-population genetic diversity of *C. tribuloides* was evaluated using various genetic indices. Expected heterozygosity (H_E_), allelic diversity, and overall genetic diversity were assessed using ARLEQUIN version 3.5.2 [17]. To further explore genetic structure, POPGENE32 software version 1.32 [19] was used to estimate key population genetic parameters, including the genetic differentiation coefficient (G_ST_), inbreeding coefficient (F_is_), and an indirect estimate of gene flow (N_m_ = 0.25(1 − F_st_)/F_st_). Additionally, potential population bottlenecks were investigated using BOTTLENECK version 1.1.03 [20], which detects reductions in population size through changes in allele frequencies and heterozygosity levels.

Inter-population genetic relationships were examined using multiple statistical approaches. Population structure and genetic variation were assessed through Analysis of Molecular Variance (AMOVA) in Arlequin 3.5.2.2 [17], allowing for partitioning of genetic variation among samples and populations. To detect cryptic population structure, Bayesian clustering analysis was performed using STRUCTURE version 2.3.4 [21,22,23], employing the admixture model with correlated allele frequencies and incorporating sampling location information via the LOCPRIOR model. The analysis included ten independent runs for each number of clusters (K) ranging from 1 to 11, with a burn-in of 100,000 iterations followed by 200,000 Markov Chain Monte Carlo (MCMC) iterations. The optimal K value was determined using the ΔK method [24], as implemented in STRUCTURE Harvester [25], while CLUMPAK [26] and DISTRUCT were used for visualization. Additionally, genetic relatedness among samples was assessed via principal component analysis (PCA), where a genetic distance matrix based on the sum of squared differences (R_st_) was generated using Arlequin and visualized in three dimensions using the R statistical software Version 4.3.2.

## 3. Results

### 3.1. STR Locus Polymorphism

Eleven STR loci were employed to assess the genetic diversity of the 84 *C. tribuloides* individuals sampled from Doi Suthep mountain, Chiang Mai, Thailand. Diversity and forensic statistical parameters for each locus are presented in Table 2. The number of alleles per locus ranged from 3 alleles for locus CT097 to as many as 23 alleles for locus CT128, demonstrating the varying levels of polymorphism across the selected markers. After applying a locus-wise Bonferroni correction (α = 0.05/11 ≈ 0.0045) to each test, nine of the eleven loci showed significant deviation from Hardy–Weinberg equilibrium (HWE), with only CT159 and CT165 conforming to HWE expectations. Using MicroChecker v2.2.3 [27], evidence of null alleles was detected at eight out of the 11 loci (CT110, CT127, CT128, CT132, CT135, CT149, CT161, CT164) after applying the Bonferroni correction (*p* < 0.0045).

Locus polymorphism was evaluated using observed (H_O_) and expected (H_E_) heterozygosity, as well as polymorphic information content (PIC). The H_O_ reached its highest value in locus CT165, while the H_E_ and PIC values were maximized in locus CT128. Conversely, locus CT161 consistently displayed the lowest values across all calculated parameters, indicating its relatively limited diversity. The power of discrimination (PD) exhibited substantial variation across loci, ranging from a minimum of 0.3075 for CT161 to a maximum of 0.9641 for CT165. Similarly, the power of exclusion (PE) and typical paternity index (TPI) showed their lowest values in CT161 (PE = 0.0139, TPI = 0.5758) and their highest values in CT165 (PE = 0.8140, TPI = 5.5000). Collectively, these eleven microsatellite markers yielded a combined matching probability (CMP) of 1 in 3.25 × 10^−8^, a combined power of discrimination (CPD) of 0.00889561, and a combined power of exclusion (CPE) of 2.24 × 10^−11^, indicating the high resolving power of this marker set for population genetic analyses.

### 3.2. C. tribuloides Population Diversity

The genetic diversity parameters varied across the three altitudinal populations of *C. tribuloides* (Table 3). The average expected heterozygosity (H_E_) values derived from the STRs analysis revealed a slight gradient, with values ranging from 0.549 in the high-altitude population to 0.565 in the low-altitude population. This suggests marginally higher genetic diversity in the low-altitude population, though the difference was relatively minor. The genetic differentiation among the populations demonstrated a moderate level, indicating some degree of genetic distinction between altitudinal groups while maintaining overall cohesion as a species.

Inbreeding appears to be a significant factor shaping the genetic structure of these populations. The inbreeding coefficient (F_is_) values were notably high, ranging from 0.243 to 0.3046 across the altitudinal gradient, and all values were statistically significant. These elevated F_is_ values indicate substantial non-random mating within the populations, suggesting potential population substructuring or other factors limiting outcrossing. Concurrent with the inbreeding findings, gene flow estimates (Nm) were consistently low across all populations, ranging from 0.1333 to 0.1528, which reflects limited genetic exchange between the altitudinal populations and supports the observed moderate genetic differentiation.

The Analysis of Molecular Variance (AMOVA) revealed the hierarchical distribution of genetic variation within and among the three altitudinal populations (Table 4). The majority of genetic variation was found within populations (17.0%), with a smaller but significant portion attributable to differences between altitudinal zones (2.83%). This pattern is consistent with the moderate genetic differentiation observed through other analyses and suggests that while gene flow is restricted, complete isolation between populations has not occurred.

### 3.3. Population Structure Analysis

**Figure 1:**
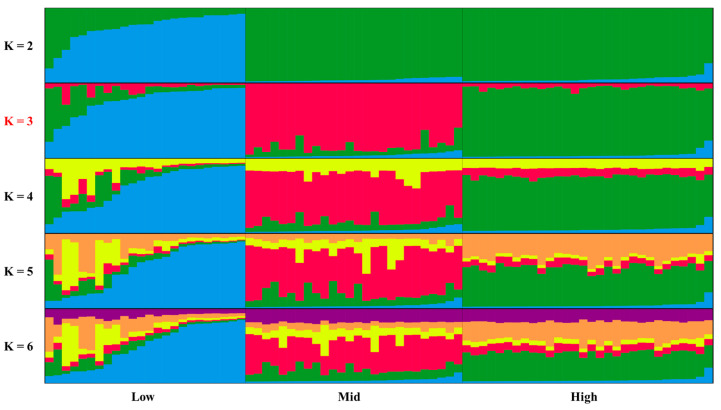
Genetic structure of the three populations of *Castanopsis tribuloides.* K 2–6 refers to genetic components. Low, mid, and high refer to elevations.

The Bayesian clustering analysis implemented in STRUCTURE, optimized using the △K method [24], identified the most likely number of genetic clusters within the sampled individuals. The STRUCTURE analysis, incorporating the LOCPRIOR model to utilize sampling location information, revealed a pattern of population substructuring that corresponded partially but not entirely with the three altitudinal zones (Figure 1). Individual membership coefficients indicated varying degrees of admixture across the identified genetic clusters, with some individuals showing strong assignment to a single cluster while others displayed mixed ancestry.

The genetic distance (R_st_) values between the three populations quantified the extent of genetic differentiation (Table 5). All pairwise R_st_ values were statistically significant (*p* < 0.05 or *p* < 0.01), confirming genuine genetic differentiation between the altitudinal populations. The magnitude of these R_st_ values aligned with the moderate genetic differentiation pattern consistently observed throughout our analyses, further supporting the conclusion that the *C. tribuloides* populations across the altitudinal gradient of Doi Suthep mountain maintain distinct genetic identities while experiencing some degree of genetic exchange.

**Figure 2:**
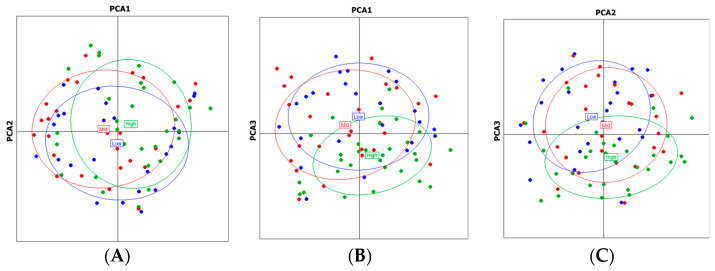
PCA analysis based on the genetic distance matrix in the three *Castanopsis tribuloides* populations (**A**): Dim.1 vs. Dim.2, (**B**): Dim.1 vs. Dim.3, (**C**): Dim.2 vs. Dim.3.

Principal component analysis (PCA) based on the genetic distance matrix (R_st_) provided complementary visualization of the genetic relationships among individuals and populations (Figure 2). The first three principal coordinates collectively explained a substantial portion of the genetic variation. In the two-dimensional PCA plots (Dim.1 vs. Dim.2, Dim.1 vs. Dim.3, and Dim.2 vs. Dim.3), individuals from the three altitudinal populations showed some clustering according to their origins, but with considerable overlap. This pattern reinforces the finding of moderate genetic differentiation with ongoing gene flow, albeit limited, between the altitudinal populations.

The bottleneck analysis, conducted under three different mutation models (infinite allele model, stepwise mutation model, and two-phase model), investigated historical changes in population size (Table 6). The results of this analysis provided insights into whether the current populations have experienced recent reductions in effective population size that could impact their genetic diversity and structure. These findings contribute to our understanding of the demographic history of *C. tribuloides* in the study area and its implications for conservation management.

## 4. Discussion

### 4.1. Genetic Diversity Patterns in C. tribuloides Populations

The present study reveals significant insights into the genetic structure of *C. tribuloides* populations across an altitudinal gradient on Doi Suthep mountain, Thailand. Our findings demonstrate moderate levels of genetic diversity within these populations, with expected heterozygosity values ranging from 0.549 to 0.565. This moderate genetic diversity is notable when compared to other *Castanopsis* species [28], indicating that *C. tribuloides* on Doi Suthep mountain maintains sufficient genetic variation, possibly for potential adaptation to environmental changes. The slightly higher genetic diversity observed in the low-altitude population compared to high-altitude populations suggests that elevation may influence genetic diversity patterns in this species. This pattern might be attributed to higher tree density, increased pollinator activity, or more favorable environmental conditions at lower elevations that facilitate greater genetic exchange. Similar altitudinal patterns of genetic diversity have been documented in other tree species, for example, the wild ancient tea tree (*Camellia taliensis*) populations at different altitudes in Qianjiazhai in China [29], where genetic diversity often decreases with increasing altitude due to harsher environmental conditions and smaller effective population sizes at higher elevations.

The significant deviation from Hardy–Weinberg equilibrium observed in nine of the eleven STR loci further supports the presence of non-random mating and population substructuring within the study area. Although null alleles were detected, the deviation from HWE, along with the high inbreeding coefficients (F_is_ = 0.243–0.3046) detected across all populations, indicates substantial inbreeding within the altitudinal zones. The elevated inbreeding levels might be explained by limited pollen dispersal distances, philopatric seed dispersal mechanisms, or vegetative reproduction strategies that are common in many forest tree species. Similar patterns of significant inbreeding have been reported in *Castanopsis sieboldii* and *C. cuspidata* populations in Japan [8], suggesting that inbreeding might be a common feature in the genus *Castanopsis*. Our findings align with previous research on tropical tree species, which often exhibit significant inbreeding despite being predominantly outcrossing, due to fine-scale spatial genetic structure resulting from limited seed dispersal.

The low levels of gene flow (N_m_ = 0.1333–0.1528) detected between the altitudinal populations further emphasize the limited genetic exchange occurring across the elevation gradient. This restricted gene flow could be attributed to phenological differences between populations at different elevations, physical barriers to dispersal, or selection pressures maintaining local adaptation. The combination of limited gene flow and significant inbreeding could potentially increase genetic drift effects and lead to further differentiation between populations over time if not counterbalanced by occasional long-distance dispersal events. These patterns of limited gene flow are frequently observed in mountainous landscapes where topographic features and environmental gradients can impede genetic exchange between populations at different elevations.

### 4.2. Population Structure Across Altitudinal Gradients

The STRUCTURE analysis revealed population substructuring that partially aligns with the altitudinal zonation, suggesting that elevation plays a role in shaping the genetic architecture of *C. tribuloides* on Doi Suthep mountain. However, the incomplete correspondence between genetic clusters and altitudinal zones indicates that other factors beyond elevation also influence the genetic structure. These factors might include historical processes, microhabitat differences unrelated to altitude, or varying selection pressures across the landscape. The moderate genetic differentiation observed among the altitudinal populations, as evidenced by significant pairwise R_st_ values, further supports the existence of distinct genetic entities while maintaining species cohesion.

The PCA results complement the STRUCTURE findings, showing some clustering according to altitudinal origins but with considerable overlap. This pattern of partial differentiation with overlap suggests a scenario in which local inbreeding and restricted gene flow are evident within and among populations at different elevations, yet the overall genetic structure remains subtle, likely reflecting a complex interplay between past gene flow and recent fragmentation. Similar patterns of incomplete genetic differentiation along altitudinal gradients have been reported in other forest tree species, reflecting the complex interplay between divergent selection pressures and homogenizing gene flow across environmental gradients.

The AMOVA results, showing that the majority of genetic variation occurs within populations rather than between them, are consistent with patterns typically observed in outcrossing tree species. This distribution of genetic variation suggests that while altitudinal adaptation is occurring, it has not yet led to strong genetic isolation between populations. The hierarchical distribution of genetic variation, with a smaller but significant portion attributable to differences between altitudinal zones, further confirms the role of elevation in structuring these populations. This genetic architecture, characterized by high within-population diversity and moderate between-population differentiation, is advantageous for adaptation to environmental heterogeneity and resilience to environmental changes.

### 4.3. Comparison with Other Castanopsis Species

The genetic diversity and population structure patterns observed in *C. tribuloides* in this study show both similarities and differences when compared to other *Castanopsis* species studied in different regions. Previous research on *C. sieboldii* and *C. cuspidata* in Japan [10] reported high genetic diversity and low genetic differentiation among populations, but with significant inbreeding coefficients similar to our findings. This suggests that significant inbreeding may be a common characteristic across the *Castanopsis* genus, possibly related to their reproductive biology or ecological characteristics. However, the moderate genetic differentiation detected in our *C. tribuloides* populations contrasts with the low differentiation reported in Japanese *Castanopsis* species, indicating potential differences in dispersal capabilities or landscape connectivity between these systems.

Studies on *C. fargesii* in China [28] revealed high genetic diversity, moderate genetic differentiation, and extensive gene flow among populations. In contrast, our *C. tribuloides* populations demonstrated moderate genetic diversity, moderate differentiation, but limited gene flow. These differences might reflect varying ecological contexts, landscape features, or dispersal mechanisms between the species. The more restricted gene flow in our study system could be related to the steeper topography and more pronounced environmental gradients on Doi Suthep mountain compared to the study sites in China.

Research on *C. eyrei* in China [11] found that genetic diversity was higher in medium-altitude populations compared to lower or higher altitudes. This differs from our finding of slightly higher genetic diversity in lower-altitude *C. tribuloides* populations. These contrasting patterns suggest that the relationship between altitude and genetic diversity may be species-specific or context-dependent, influenced by local environmental conditions, population history, or species-specific life-history traits. The varying responses to altitudinal gradients across *Castanopsis* species highlight the importance of species-specific studies for understanding genetic patterns and informing conservation strategies.

### 4.4. Bottleneck Analysis and Historical Population Dynamics

The bottleneck analysis conducted under different mutation models provides insights into the historical demographic changes in *C. tribuloides* populations. The detection of bottleneck signatures suggests that these populations may have experienced past reductions in effective population size, potentially due to historical climatic events, anthropogenic disturbances, or natural disasters. Understanding these historical population dynamics is crucial for interpreting current genetic patterns and predicting future population trajectories. Recent studies on other tree species have demonstrated that historical bottlenecks can have long-lasting effects on genetic diversity, often visible long after population sizes have recovered.

The detection of bottlenecks across multiple altitudinal zones suggests that historical events affecting population sizes likely impacted the entire Doi Suthep mountain region rather than being localized to specific elevations. This broad-scale impact could be related to regional climatic fluctuations, perhaps associated with Quaternary climate oscillations that affected many Southeast Asian forests. Alternatively, anthropogenic disturbances that have occurred throughout the region’s history could have caused population reductions across all elevations. Historical bottlenecks have been documented in numerous plant species throughout Southeast Asia, often attributed to Pleistocene climate changes or more recent human activities.

## 5. Conclusions

The findings of this study have important implications for the conservation and management of *C. tribuloides* on Doi Suthep mountain and potentially throughout its range. The moderate genetic diversity, significant inbreeding, and limited gene flow observed across the altitudinal gradient suggest that these populations may be vulnerable to further fragmentation or environmental changes. Conservation strategies should aim to maintain connectivity between altitudinal zones to facilitate gene flow and prevent further genetic isolation, which could exacerbate inbreeding and reduce adaptive potential.

The genetic distinctiveness of populations across the altitudinal gradient indicates that conservation efforts should encompass the full range of elevations to preserve the species’ total genetic diversity. This approach aligns with the growing recognition that conserving genetic diversity across environmental gradients is essential for maintaining species’ adaptive potential in the face of climate change. For ex situ conservation programs, sampling should include individuals from all altitudinal zones to capture the full spectrum of genetic variation present in the species.

The detection of bottleneck signatures across populations suggests that *C. tribuloides* may have experienced historical reductions in effective population size, potentially making them more vulnerable to current and future threats. Restoration efforts should consider using diverse genetic material from multiple source populations to enhance genetic diversity and adaptive potential in restored populations. Additionally, the monitoring of genetic diversity over time would be valuable for assessing the effectiveness of conservation interventions and detecting any further genetic erosion.

While this study provides valuable insights into the genetic structure of *C. tribuloides* populations, several limitations should be acknowledged. The sampling was confined to Doi Suthep mountain, and, therefore, the findings may not be representative of the species’ genetic patterns throughout its entire range. Future studies should expand sampling to include populations from different mountain ranges and geographic regions to provide a more comprehensive understanding of the species’ genetic diversity and structure at broader spatial scales.

The use of neutral STR markers in this study provides information about genetic diversity and structure; however, it does not directly assess adaptive genetic variation. Moreover, the detection of null alleles, often caused by mutations at primer binding sites, in most of the STR loci raises the possibility that the studied set of STR markers may not be fully compatible with the *C. tribuloides* genomic background. Future research incorporating genomic approaches, such as genome-wide association studies or environmental association analyses, would be valuable for identifying potential adaptive loci responding to altitudinal gradients or other environmental factors. Such studies could provide deeper insights into the genetic basis of adaptation to different elevations and the potential for adaptive responses to climate change.

The current study focused primarily on spatial patterns of genetic variation but did not include temporal sampling to assess changes in genetic diversity over time. Long-term monitoring of genetic diversity in these populations would be valuable for detecting potential genetic erosion, evaluating the impacts of environmental changes, and assessing the effectiveness of conservation interventions. Additionally, integrating genetic data with ecological and phenotypic data would provide a more comprehensive understanding of how genetic variation correlates with adaptive traits and ecological performance across environmental gradients.

Future research should also investigate the reproductive biology, mating system, and dispersal mechanisms of *C. tribuloides* in more detail to better understand the processes driving the observed genetic patterns. Studies on pollen and seed dispersal distances, patterns of pollen flow, and fine-scale spatial genetic structure would provide valuable insights into the mechanisms underlying the significant inbreeding and limited gene flow detected in this study. Furthermore, comparative studies across multiple *Castanopsis* species would help identify common patterns and species-specific responses, enhancing our understanding of the evolutionary and ecological processes shaping genetic diversity in this important genus of Asian forests.

## Figures and Tables

**Table 1:** Characteristics of eleven STR primers developed for *Castanopsis tribuloides*.

**Primer**	SSR Motif	Primer Sequence (5′–3′)		Amplification Size Range (bp)	Tm (°C)
CT097	(ATT)n	F: PET-CGACTTTGGGAAGGAAATAAAGG	R: TGGACTTCAACTTGCCATAGTG	139–175	55
CT110	(TGT)n	F: PET-TTCTTCAGTTAGCCACATCG	R: CGCTAAGTCCATACATACAACAG	169–205	55
CT127	(AGA)n	F: TAMRA- CCAGAAAACGTATGATCTTTG	R: CCATGCAACACTACCTCGTC	207–246	62
CT128	(TCA)n	F: TAMRA- CCTTGGCAGACAAACTAGATA	R: GGCGCAACAACATATGAAGAAT	160–244	56
CT132	(AT)n(AAG)n	F: TAMRA-TGACCCGAGCATGGTTTAT	R: GGACGTTAGGCCTGTACATT	126–174	56
CT135	(TGA)n(GAA)n	F: FAM-GCCTAGCTTATGGAGTGGTT	R: GTCTTTGTGCAAGTGCTC	124–160	55
CT149	(TCT)n	F: FAM-GCGCGTGACTTAGGCTCTTCAC	R: CTTCTCTGTTGGCATTTCTTGC	144–150	56
CT159	(TCT)n	F: FAM- ATCCATGTCCACTTCTTCAA	R: CGTTTCCAAAACGAAGAAC	130–193	55
CT161	(CAC)n	F: PET-AACGATACTAGCGACCTTGA	R: GCGAAAAACGCTCTCCAAC	136–166	56
CT164	(CAC)n	F: HEX-ACAACACACCTAACATCACAAC	R: GAATGTTGCTCAGCGAAG	124–174	55
CT165	(CTT)n	F: HEX-AGCGCCTTCTTAATAGAACC	R: TGGTGACCATTACTTGTTGA	112–149	55

**Table 2:** Microsatellite primers used to assess genetic diversity in 84 *Castanopsis tribuloides* individuals from Doi Suthep, Chiang Mai, Thailand.

	Na	Ho	He	PD	MP	PIC	PE	TPI	P_HWE_	Null Allele
CT097	3	0.4375	0.3768	0.5194	0.4806	0.3131	0.1384	0.8889	0.0008	no
CT110	9	0.2405	0.3164	0.4509	0.5491	0.3026	0.0418	0.6583	0.0000	yes
CT127	13	0.5417	0.6593	0.8044	0.1956	0.6313	0.2266	1.0909	0.0000	yes
CT128	23	0.4533	0.9310	0.9614	0.0386	0.9200	0.1498	0.9146	0.0000	yes
CT132	10	0.4359	0.7465	0.8583	0.1417	0.7067	0.1373	0.8864	0.0000	yes
CT135	9	0.6027	0.7850	0.9022	0.0978	0.7511	0.2942	1.2586	0.0000	yes
CT149	6	0.3924	0.4754	0.6662	0.3338	0.4467	0.1094	0.8229	0.0009	yes
CT159	6	0.2308	0.2665	0.4270	0.5730	0.2526	0.0387	0.6500	0.1052	no
CT161	6	0.1316	0.2094	0.3075	0.6925	0.2030	0.0139	0.5758	0.0006	yes
CT164	8	0.2949	0.6099	0.7521	0.2479	0.5305	0.0615	0.7091	0.0000	yes
CT165	15	0.9091	0.8883	0.9641	0.0359	0.8717	0.8140	5.5000	0.3610	no

Na, number of alleles; Ho, observed heterozygosity; He, expected heterozygosity; GD, gene diversity; PD, power of discrimination; MP, matching probability; PIC, polymorphic information content; PE, power of exclusion; TPI, typical paternity index (TPI); null allele: sign of null allele detection.

**Table 3:** Expected heterozygosity (H_E_), gene diversity (GD), genetic differentiation coefficient (G_ST_), inbreeding coefficient (F_is_), and indirect estimate of gene flow (N_m_) of *C. tribuloides* from different altitudes on Doi Suthep mountain, Chiang Mai, Thailand.

	He	GD	G_ST_	Fis	Nm
Low elevation (n = 24)	0.565	0.545	0.6207	0.2413	0.1528
Mid elevation (n = 30)	0.561	0.344	0.6523	0.3046	0.1333
High elevation (n = 30)	0.549	0.511	0.6477	0.2954	0.1360

**Table 4:** Analysis of Molecular Variance (AMOVA) between the three populations of *Castanopsis tribuloides*.

Source of Variation	Sum of Squares	Percentage of Variation	Statistics	*p* Value *
Among population	9.889	2.83	*F_ST_* = 0.02826	0
Among individual within population	164.498	17	*F_IS_* = 0.175	0.0059
Within individuals	120	80.17	*F_IT_* = 0.19831	0

* Significance of test was based on 1000 permutations.

**Table 5:** Genetic distance (*R_st_*) values (below diagonal) and *p*-value of genetic distance (above diagonal) of three *Castanopsis tribuloides* populations.

	Low Elevation	Mid Elevation	High Elevation
Low elevation	0	0.0078	0.0166
Mid elevation	0.0323 **	0	0.0010
High elevation	0.0256 *	0.0395 **	0

* = *p* < 0.05, ** = *p* < 0.01.

**Table 6:** STR bottleneck events.

Population	Sign Test			Standardized			Wilcoxon Test			Allele Frequency Distribution
	IAM	TPM	SMM	IAM	TPM	SMM	IAM	TPM	SMM	
Low	0.1233	0.0343	0.0009	0.1228	0.0001	0.0000	0.8608	0.9919	0.9995	L-shaped
Mid	0.1509	0.0098	0.0107	0.0739	0.0001	0.0000	0.8799	0.9939	0.9976	L-shaped
High	0.4524	0.5214	0.0013	0.2731	0.0043	0.0000	0.5508	0.8174	0.9993	L-shaped

Infinite allele model (IAM), stepwise mutation model (SMM), and two-phase model (TPM).

## Data Availability

The original contributions presented in this study are included in the article. Further inquiries can be directed to the corresponding authors.
